# Predicting sarcopenia risk in stroke patients: a comprehensive nomogram incorporating demographic, anthropometric, and biochemical indicators

**DOI:** 10.3389/fneur.2024.1438575

**Published:** 2024-12-09

**Authors:** Yufan Pu, Ying Wang, Huihuang Wang, Hong Liu, Xingxing Dou, Jiang Xu, Xuejing Li

**Affiliations:** The Affiliated Huai'an Hospital of Xuzhou Medical University, Huai'an, Jiangsu, China

**Keywords:** sarcopenia, post-stroke, nomogram, risk, biochemical, hematological

## Abstract

**Objective:**

Although there is a strong correlation between stroke and sarcopenia, there has been a lack of research into the potential risks associated with post-stroke sarcopenia. Predictors of sarcopenia are yet to be identified. We aimed at developing a nomogram able to predict sarcopenia in patients with stroke.

**Methods:**

The National Health and Nutrition Examination Survey (NHANES) cycle year of 2011 to 2018 was divided into two groups of 209 participants—one receiving training and the other validation—in a random manner. The Lasso regression analysis was used to identify the risk factors of sarcopenia, and a nomogram model was created to forecast sarcopenia in the stroke population. The model was assessed based on its discrimination area under the receiver operating characteristic curve, calibration curves, and clinical utility decision curve analysis curves.

**Results:**

In this study, we identified several predictive factors for sarcopenia: Gender, Body Mass Index (kg/m^2^), Standing Height (cm), Alkaline Phosphatase (ALP) (IU/L), Total Calcium (mg/dL), Creatine Phosphokinase (CPK) (IU/L), Hemoglobin (g/dL), and Waist Circumference (cm). Notably, female patients with stroke exhibited a higher risk of sarcopenia. The variables positively associated with increasing risk included Alkaline Phosphatase, Body Mass Index, Waist Circumference, and Hemoglobin, while those negatively associated with risk included Height, Total Calcium, and Creatine Phosphokinase. The nomogram model demonstrated remarkable accuracy in distinguishing between training and validation sets, with areas under the curve of 0.97 and 0.90, respectively. The calibration curve showcased outstanding calibration, and the analysis of the decision curve revealed a broad spectrum of beneficial clinical outcomes.

**Conclusion:**

This study creates a new nomogram which can be used to predict pre-sarcopenia in stroke. The new screening device is accurate, precise, and cost-effective, enabling medical personnel to identify patients at an early stage and take action to prevent and treat illnesses.

## Introduction

Sarcopenia, a musculoskeletal condition defined by the progressive loss of muscle mass and strength (1), particularly in elderly populations, is a phenomenon that has established itself as a significant medical issue (2). This disorder results in adverse consequences, including falls, functional decline, frailty, and mortality (3). Therefore, it is pivotal to understand sarcopenia's risk factors, strategies to cope, and potential treatments, thereby intensifying the importance of extensive research on this topic. To comprehend the significance of sarcopenia research, one must view this condition not merely as an individual health concern but indeed as a global issue (4). The diagnosis of sarcopenia encompasses decreased levels of muscle strength, muscle quantity or quality, and physical performance. Such musculoskeletal degeneration impairs daily activities and poses a real threat to individual autonomy. However, the disorder's full scope extends beyond the personal level, potentially straining healthcare systems due to the increased burden of care for the elderly (5). Hence, sarcopenia research is vital in mitigating these issues. Stroke and sarcopenia constitute two significant health issues with substantial impacts on older adults' health and quality of life (6). Dynamic is the relationship between stroke and sarcopenia—the loss of muscle mass and strength—commonly seen in the elderly population (7). Despite the growing body of scientific literature exploring these two conditions separately, comprehensive research examining their interplay is needed to fully grasp how they influence one another (8–10). Stroke often leads to varying degrees of disability due to motor impairments and can interfere with a patient's functional ability, both of which can exacerbate sarcopenia's progression (11). This sarcopenic progression following stroke has been associated with poorer recovery outcomes and increased disability (12). Substantial attention should be directed toward the stroke and sarcopenia relationship to fully understand and effectively address these underlying mechanisms (11). Patients who experienced stroke often exhibit significant muscle changes, including muscle atrophy and increased intramuscular fat on the side of the body affected by the stroke (13). Research by Aydin et al. (14) indicates that these muscle alterations might serve as a link to post-stroke sarcopenia and reinforces the importance of further research. Given that sarcopenia is characterized by functional deficits, it can substantially interfere with post-stroke rehabilitation (8). Limited muscle strength and mass may negatively influence a patient's ability to participate in rehabilitation activities, ultimately affecting recovery outcomes (15).

Recent research has indicated that sarcopenia can be reversed or prevented, implying that stroke patients suffering from sarcopenia are likely to experience positive outcomes from timely diagnosis and intervention, particularly during the initial phase of sarcopenia (16). Nevertheless, the assessment of skeletal muscle mass is exceedingly restricted, necessitating the utilization of specialized apparatus like dual-energy X-ray absorptiometry (DXA), bio-impedance analysis (BIA), X-ray computed tomography (CT), or magnetic resonance imaging (MRI) (17).

In recent years, predictive models have been developed to assess the risk of sarcopenia in general populations, often leveraging demographic, biochemical, and hematological parameters (18). Nomograms, for instance, have become increasingly valuable as visual prediction tools, translating complex statistical models into practical applications for clinicians (19). These models have demonstrated accuracy in estimating sarcopenia risk and guiding early interventions in general healthcare settings (20, 21).

However, in the context of stroke patients, research on sarcopenia prediction is relatively sparse, despite the high prevalence and impact of sarcopenia in this population. Stroke survivors are at particular risk due to the compounded effects of neurological impairments and prolonged immobility, which can accelerate muscle atrophy (22). While some studies have examined predictors of functional decline post-stroke (23), few have applied predictive modeling approaches, such as nomograms, specifically tailored to forecast sarcopenia risk in this group (24, 25). This gap indicates a need for targeted tools that could support timely interventions in stroke recovery settings.

In light of this, our study aimed to develop and validate a nomogram to predict sarcopenia risk in stroke patients using data from the National Health and Nutrition Examination Survey (NHANES). This tool incorporates key predictors identified through Lasso regression analysis, enabling clinicians to assess sarcopenia risk more effectively and personalize care in post-stroke management. The hypothesis of this study is that in stroke patients, sarcopenia risk can be effectively predicted based on specific demographic and biochemical indicators using a constructed nomogram model.

## Methods

### Data source and study population

**Figure 1:**
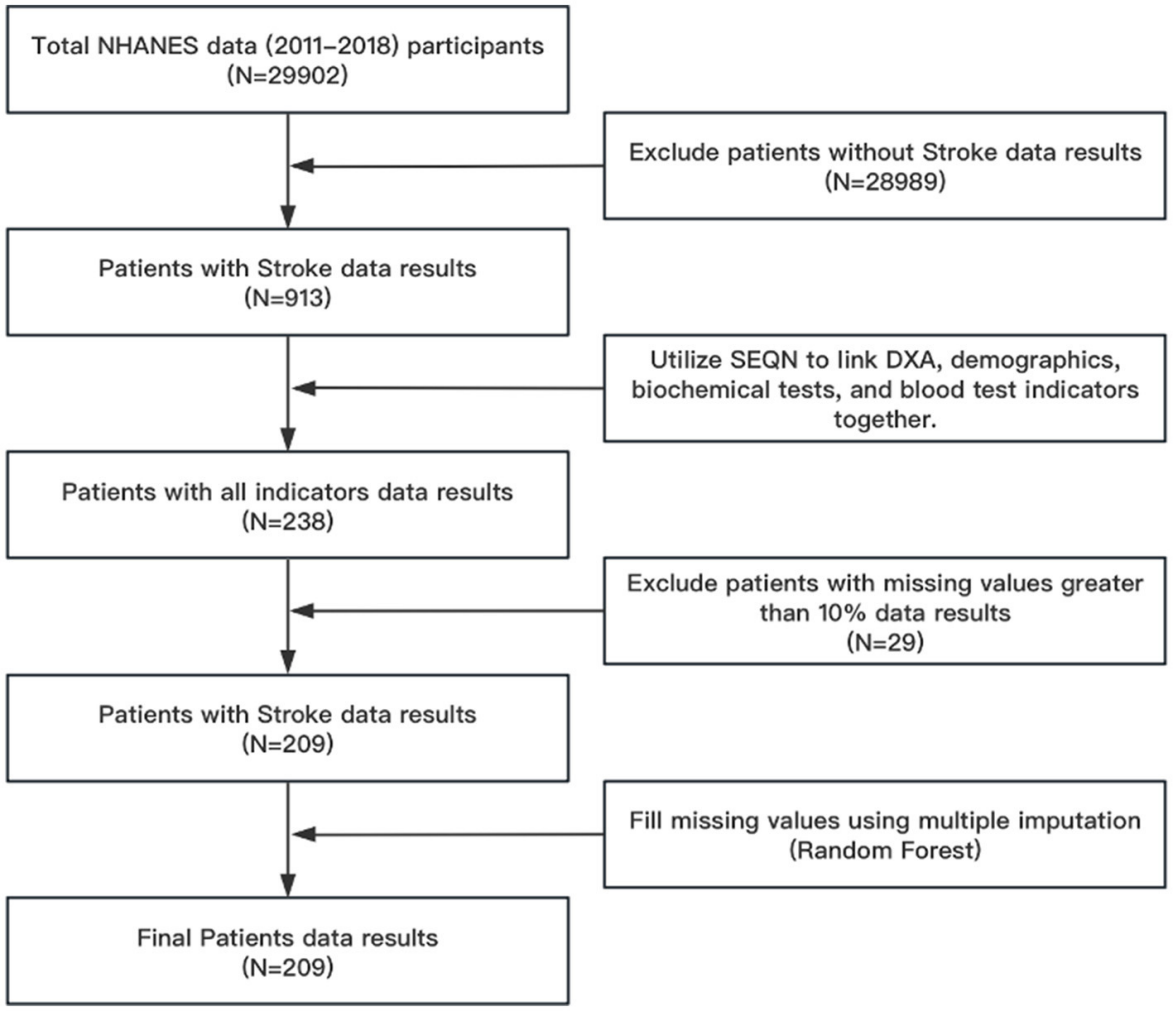
Flow chart for screening research samples. NHANES, National Health and Nutrition Examination Survey; DXA, Dual energy x-ray absorptiometry; SEQN, Respondent sequence number.

In this study, we gathered data from NHANES, a comprehensive survey conducted by the esteemed National Center for Health Statistics (NCHS), which falls under the Centers for Disease Control and Prevention (CDC). It aims to assess the health and nutritional status of adults and children in the United States. NHANES uses a complex, multistage, probability sampling design to select a representative sample of about 5,000 participants each year from 15 counties across the country. The flowchart for the research is shown in Figure 1. From 2011 to 2018, the NHANES data encompassed a total of 29,902 individuals. With the exception of individuals who did not experience a stroke, a total of 913 individuals remained. The number of people linked using Respondent sequence number (SEQN) is 238 because there are fewer people with laboratory examinations in the NHANES database than those with questionnaires. After removing samples with a missing rate of more than 10%, 209 individuals were still present. Due to the fact that removing all samples with missing values will render the research unfeasible, multiple imputation techniques are employed to complete missing values for samples with fewer missing values. The effectiveness of random forests in multiple imputation methods has been confirmed by studies (26, 27), so this article uses multiple imputation methods. Utilizing a random forest methodology for interpolation. The process is shown in Figure 1.

### Measurements and definition of sarcopenia

The NHANES dataset utilized DXA to measure body composition, employing the Hologic QDR-4500 A fan beam densitometer, a dependable device produced by Hologic, Inc., situated in Bedford, MA, USA. To acquire a thorough collection of DXA findings, we amassed data files encompassing the period from 2011 to 2018 within NHANES. The metric of appendicular skeletal muscle mass (ASM), which measures the combined lean mass of the arms and legs, is widely accepted in clinical practice. In order to carry out our analysis, we utilized the skeletal muscle mass index (SMI), a metric endorsed by the Foundation for the National Institutes of Health (FNIH) Sarcopenia. SMI entails modifying ASM based on body mass index (BMI) (28). Our research revealed that men with a SMI of <0.789 or women with a SMI of <0.512 had a low muscle mass, thus satisfying the criteria for sarcopenia (29).

### Procedure

The dataset was partitioned into training and validation sets using a random allocation method, ensuring an equitable split of 5:5 proportions. The judgment of stroke in patients in this study comes from a questionnaire in the NHANES database. The question is “Has a doctor or other health professional ever told {you/SP} that {you/s/he}...had a stroke?” Additional variables include gender, age, BMI, height, weight, waist circumference, race, complete blood cytology, and biochemical tests. Race is divided into four categories: Mexican American; non-Hispanic white; non-Hispanic black, and others. BMI is defined as body weight (kilograms) divided by height (meters) squared.

### Statistical analysis

The descriptive statistics included both continuous and categorical variables. The continuous variables were subjected to group comparisons using either the *t-*test or the Wilcoxon rank-sum test. The chi-square test and Fisher's exact test were used to compare the categorical variables. Initially, the predictors underwent preliminary screening using LASSO regression in the development set. The LASSO analysis reduced the regression coefficient of variables to zero through the implementation of a penalized coefficient of Lambda. It disregarded variables that had no regression coefficients and chose variables that had no regression coefficients. The variables that were chosen were found to have the strongest association with post-stroke sarcopenia. Subsequently, the training set was used to create a prediction model through multivariate logistic regression analysis. The model was used to determine the score of each predictor. The model was visualized through the use of a nomogram. Lastly, the receiver operating characteristic (ROC) curve analysis was used to assess model discrimination. Area under the curve (AUC) values of 0.75 or higher were indicative of strong discrimination (30). The accuracy of the prediction was evaluated through the use of calibration plots. Calibration plots are a valuable tool for evaluating the accuracy of predictive models, including nomograms. They help determine how well predicted probabilities align with actual observed outcomes. A well-calibrated model will provide predicted probabilities that closely match the actual incidence of the event of interest (31). Decision curve analysis (DCA) was utilized to estimate the clinical utility (32). The test set was used to validate the nomogram. All tests employed a two-tailed approach, with a *p-*value of 0.05 or less indicating statistical significance. We used R statistical software (version 4.3.2) to carry out statistical analysis, and the nomogram was created with the help of the “nomogramFormula” package, authored by Zhi, J and Jing, Z.

## Results

### Patient characteristics

In this study, 209 people were included, with 33 having sarcopenia after stroke and 176 having non-sarcopenia after stroke, making up 15.8% of the total. The mean age of patients without sarcopenia (48.7 + 9.54) and those with sarcopenia (51.4 + 7.69) exhibited no significant statistical difference. Out of the non-sarcopenic population, 41.5% were female and 58.5% were male, whereas in the sarcopenic population, 42.2% were female and 57.6% were male. The sarcopenic and non-sarcopenic groups significant statistical differences in other demographic attributes such as race, BMI, weight, height, and waist circumference. Furthermore, Table 1 displays Baseline characteristics, and the biochemical analysis and a comprehensive array of blood test markers showed in the Supplementary material.

**Table 1:** Baseline characteristics of study patients with stroke.

**Variable**	**Non sarcopenia**	**Sarcopenia**	**P**
	***N = 176***	***N = 33***	
Gender			1.000
Male	73 (41.5%)	14 (42.4%)	
Female	103 (58.5%)	19 (57.6%)	
Age	48.7 (9.54)	51.4 (7.69)	0.074
Race			0.003
Mexican American	18 (10.2%)	5 (15.2%)	
Other Hispanic	9 (5.11%)	6 (18.2%)	
Non-Hispanic White	62 (35.2%)	16 (48.5%)	
Non-Hispanic Black	69 (39.2%)	4 (12.1%)	
Other race	18 (10.2%)	2 (6.06%)	
BMI	30.1 (7.07)	37.7 (9.44)	<0.001
Weight	85.6 (22.8)	97.3 (27.1)	0.025
Height	168 (8.59)	161 (9.54)	<0.001
WAIST	103 (17.2)	116 (16.7)	<0.001

### Predictors of post-stroke sarcopenia

**Figure 2:**
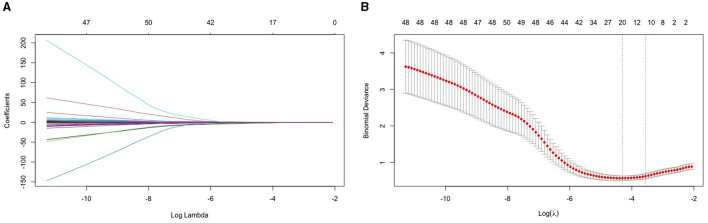
LASSO regression model for 64 variables. **(A)** The figure illustrates the variation in coefficient for each variable. The coefficient value is represented by the ordinate, while the lower abscissa corresponds to log (λ), and the upper abscissa signifies the count of non-zero coefficients in the current model. **(B)** After performing a 10-fold cross-cross validation fitting, the model was chosen. The best option is to select the lowest value with a standard deviation of one.

This study established a LASSO regression model for 68 variables screened from the NHANES database. Variables were centralized and normalized by 10-fold cross-validation. According to Figure 1, we filter variables based on the binomial deviance of log (λ). Selected predictors were Gender, Race, Body Mass Index (kg/m**^2^), Standing Height (cm), Alkaline Phosphatase (ALP) (IU/L), Total Calcium (mg/dL), Creatine Phosphokinase (CPK) (IU/L), Hemoglobin (g/dL), and Waist Circumference (cm). Secondly, to avoid the curse of dimensionality in the model, we eliminated the race variable and included the remaining eight variables to build a multiple logistic regression model (Figure 2).

### Nomogram in patients with post-stroke sarcopenia

**Figure 3:**
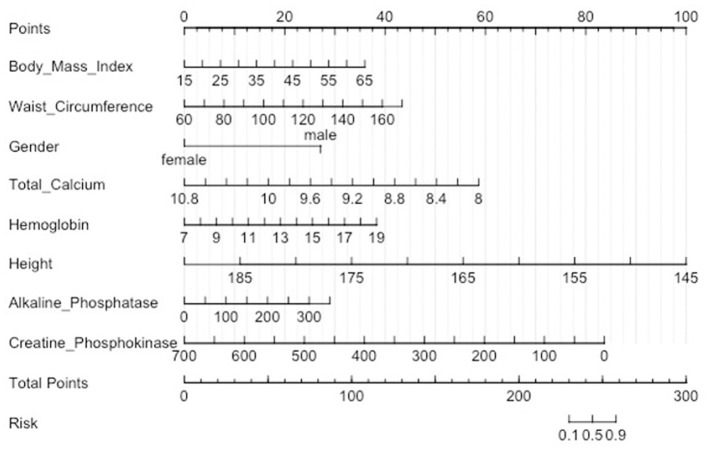
Nomogram for predicting sarcopenia in stroke patients. A certain score is indicated by each level of predictor. The score of each predictor was summarized to generate a total score. The overall score aligns with the likelihood of sarcopenia.

A nomogram was constructed to predict the risk of sarcopenia in patients with stroke. This model contained eight predictors: Gender, Body Mass Index (kg/m^**^2), Standing Height (cm), Alkaline Phosphatase (ALP) (IU/L), Total Calcium (mg/dL), Creatine Phosphokinase (CPK) (IU/L), Hemoglobin (g/dL), and Waist Circumference (cm) (Figure 3). For example, A man who is 1.55 meters tall has a BMI of 27 and a waist circumference of 110 centimeters. His blood laboratory tests were Total Calcium 8.9 mg/dL, Hemoglobin 16 g/dL, Alkaline Phosphatase 300 IU/L, and Creatine Phosphokinase 600 IU/L. The corresponding score of each predictor was 10 points, 15 points, 29 points, 40 points, 30 points, 78 points, 25 points, and 13 points respectively. His total score was 250 points. It indicated that the risk of sarcopenia was 70% in patients with stroke.

### Performance and validation of the nomogram

**Figure 4:**
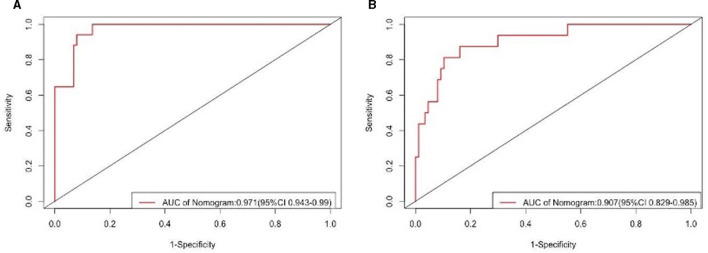
ROC curve and AUC of the predictive model. **(A)** The ROC in the development set. **(B)** The ROC in the validation set. ROC, receiver operating characteristic; AUC, area under the curve.

**Figure 5:**
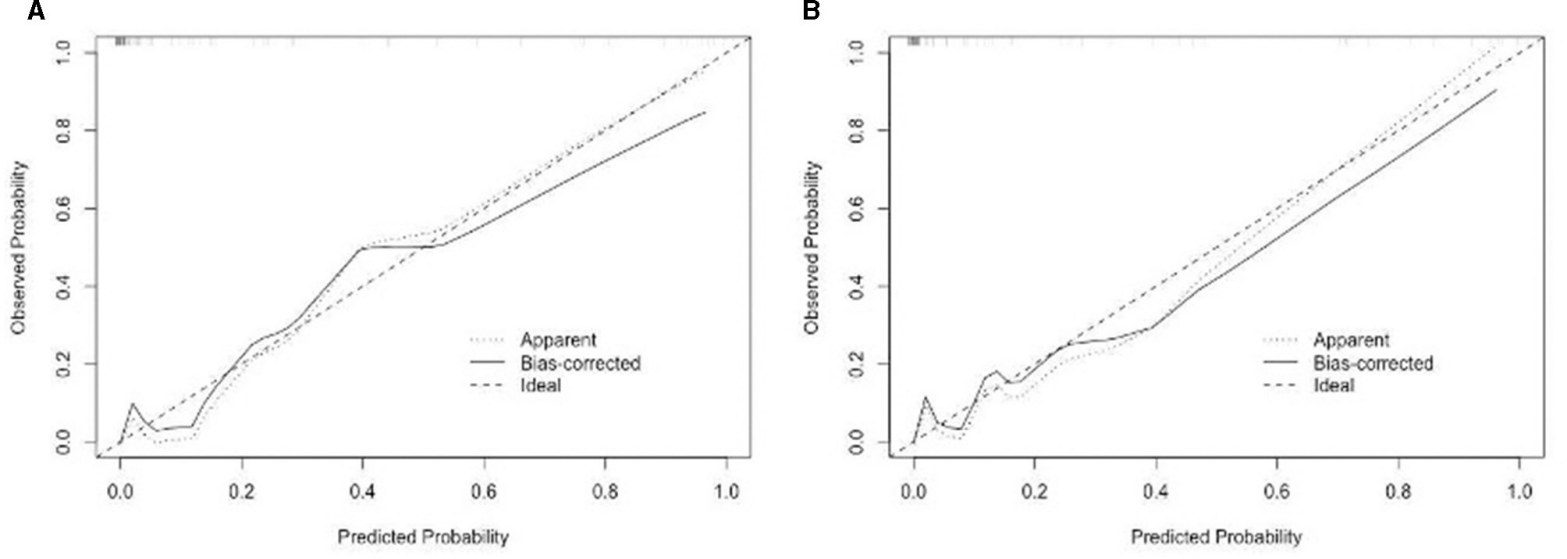
**(A)** The nomogram for sarcopenia in the training set was calibrated using a curve. **(B)** The nomogram for sarcopenia in the validation set was calibrated using a curve. The X-axis depicts the anticipated likelihood, while the Y-axis signifies the factual ratio. The ideal model's best prediction is denoted by the diagonal dotted line. The nomogram's uncorrected performance is depicted by the apparent line, whereas the bias-corrected performance is depicted by the solid line.

**Figure 6:**
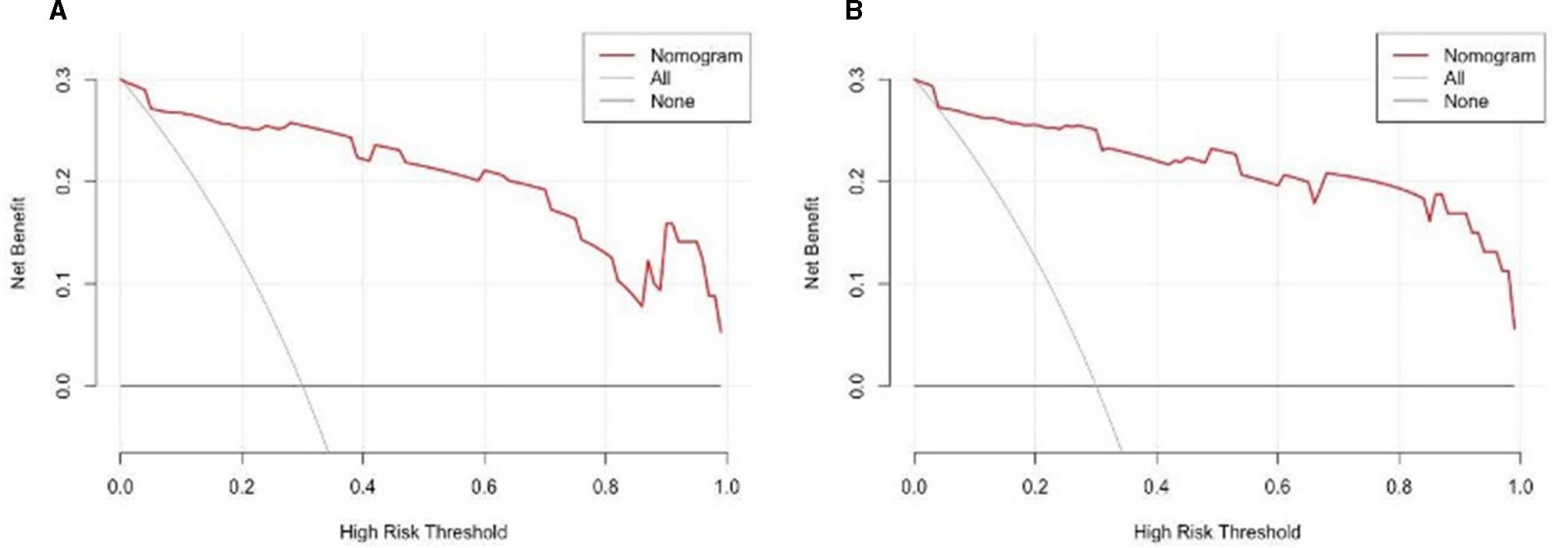
DCA of the nomogram. **(A)** The train set was occupied by the DCA. **(B)** The validation set includes DCA. The patient refrains from applying the nomogram, resulting in no net benefit, as depicted by the black-solid line; conversely, the gray-solid line indicates that all patients receive treatment based on the nomogram. The three lines enclosing the area demonstrate the practicality of the nomogram. Examining the Decision Curve of the Decision-Making Process.

The findings indicated that the anticipated results closely aligned with the observed outcomes. The ROC curve in the training set exhibited a strong ability to distinguish (AUC: 0.97; 95% CI: 0.94–0.99) (Figure 4A). The model's ability to discriminate was confirmed in the test set (0.90; 0.82–0.98) (Figure 4B). Additionally, the calibration curve analysis revealed a strong correlation between the anticipated probabilities and the observed sarcopenia after stroke in both the training and test sets (Figure 5). DCA demonstrated the clinical usefulness of this model (Figure 6).

## Discussion

This study aimed to develop a nomogram to predict sarcopenia risk in post-stroke patients using demographic and biochemical factors. Key findings identified eight predictive factors: Gender, BMI, Standing Height, ALP, Total Calcium, CPK, Hemoglobin, and Waist Circumference. The model demonstrated high accuracy, with AUC values of 0.97 and 0.90 for the training and validation sets, respectively, showing strong predictive power. Calibration and decision curve analyses further supported the model's reliability and clinical utility, suggesting it can aid in early sarcopenia intervention for stroke patients.

Personalized patient management in post-stroke care represents a core aspect of modern healthcare, particularly in the context of comorbidities such as sarcopenia, a progressive skeletal muscle disorder involving the accelerated loss of muscle mass and function (33). Current research on the development of a nomogram prediction model for sarcopenia risk in hemodialysis patients indicates promising avenues for post-stroke patients (34). The development of a nomogram predicting the risk of sarcopenia introduces a mathematical model than enhances clinical decision-making (35). This prediction model incorporates variables such as age, C-reactive protein, serum phosphorus, BMI, and mid-upper arm muscle circumference, thus offering a multidimensional risk assessment for sarcopenia in post-stroke patients (34). Such comprehensive data can aid in the early identification of high-risk patients, thus enhancing proactive patient management and potentially mitigating adverse outcomes. The introduction of a nomogram prediction model for post-stroke sarcopenia presents a potential paradigm shift in patient management. Early risk stratification and identification of sarcopenia can lead to early interventions, reducing the major complications associated with sarcopenia and improving stroke recovery outcomes (36). Further empirical research examining the statistical and clinical validity of the nomogram prediction model for post-stroke sarcopenia will offer significant contributions to stroke management. By aligning clinical practice with personalized medicine insights, we can optimize post-stroke patient outcomes, particularly concerning comorbid sarcopenia.

These Asian studies have often found high rates of sarcopenia linked to lower physical activity levels, malnutrition, and metabolic factors, such as low serum albumin and hemoglobin, particularly among post-stroke patients. This aligns with our findings where factors like Hemoglobin and biochemical markers (e.g., ALP, CPK) were significant predictors of sarcopenia. Asian research has also identified sarcopenic obesity, which reflects the complex interaction between high BMI and muscle loss—similarly seen in our study where BMI was a predictor despite obesity traditionally being a protective factor (37).

However, specific gender-related factors influencing the development or progress of this condition remain a topic of interest. Due to gender-based differences in muscle mass and hormonal influences, the overall natural history of sarcopenia may vary (33). This discrepancy emphasizes the necessity to examine gender-based differences in the incidence and progression of post-stroke sarcopenia. To address gender-based hormonal differences in sarcopenia, researchers and clinicians should adopt gender-specific approaches. Prediction models should use gender-specific baselines for muscle mass and strength, and clinical evaluations may include hormone monitoring to understand individual risk better. Tailored exercise and nutrition programs—resistance training for men and estrogen-supportive interventions for postmenopausal women—can address these differences effectively. Additionally, more gender-specific longitudinal studies are needed, and clinician education should focus on recognizing and addressing these disparities, ensuring more personalized and effective sarcopenia management. Recognizing these differences can potentially foster enhanced rehabilitation strategies, promoting improved outcomes in stroke patients. This study confirms that gender is the main factor influencing the occurrence of sarcopenia in post-stroke patients.

Contrary to the notion that higher BMI corresponds to a lower risk of sarcopenia, findings propose the existence of sarcopenic obesity, suggesting the complex interplay between BMI and sarcopenia (38). The concept of sarcopenic obesity highlights a paradox where individuals have both high body mass index (BMI) and low muscle mass (39). This can be explained by the fact that BMI does not differentiate between muscle and fat, meaning a person can appear to have adequate or excess weight while actually experiencing muscle depletion (40). Sarcopenic obesity often involves a high proportion of body fat with reduced muscle mass and quality, impacting physical function and metabolic health (41). In stroke patients, sarcopenic obesity is particularly relevant (42). Stroke survivors often experience decreased mobility, leading to muscle wasting and increased fat accumulation due to prolonged physical inactivity and metabolic disruptions (43, 44). Additionally, neurological impairments can hinder rehabilitation efforts, compounding muscle atrophy while promoting weight gain if caloric intake remains the same or increases due to stress-related eating patterns (45, 46). This combination may contribute to poor outcomes, as muscle loss impairs mobility and recovery, while excess fat increases the risk of cardiovascular complications and insulin resistance. These findings have implications for interpreting our results, as BMI alone may mask the underlying muscle loss in post-stroke patients. In our study, higher BMI was a predictor of sarcopenia, likely reflecting sarcopenic obesity. This reinforces the need to assess both muscle and fat composition in clinical settings to accurately evaluate sarcopenia risk in stroke patients, as BMI alone could misclassify at-risk individuals. Recognizing sarcopenic obesity allows for a more comprehensive approach to managing post-stroke recovery by targeting both muscle preservation and body composition improvements through tailored interventions.

While height's influence on post-stroke sarcopenia remains unclear, it forms part of the sarcopenia diagnosis, with height-based cut-off points established for muscle mass (47).

Waist Circumference, a proxy for abdominal adiposity, seems contradictory in the context of sarcopenia, with some research showing no significant association (38). Recognizing the associations between these physiological parameters and post-stroke sarcopenia may enhance our understanding of sarcopenia's multifaceted nature and broaden the horizon for therapeutic interventions. Future research should address these variables in a combined or sequential manner to uncover the overlapping and unique contributions each makes toward post-stroke sarcopenia.

The regulation of ALP, Total Calcium, CPK, and Hemoglobin within the body may influence post-stroke sarcopenia development. While aberrant ALP levels mark liver damage or bone disorders (48), their role in sarcopenia remains unexplored. Studies indicate that calcium signaling has a key role in muscle function (49), potentially influencing sarcopenic progression. Similarly, CPK, a measure of muscle destruction, may correlate with post-stroke sarcopenia (50). Lastly, Hemoglobin, indicative of anemia status, may affect sarcopenia as muscle oxygenation can impact muscle function (51).

This study presents several notable strengths. Firstly, the development of a nomogram for predicting sarcopenia in stroke patients fills a critical gap in the existing literature, addressing the correlation between these two conditions. The use of the NHANES dataset enhances the generalizability of the findings, as it reflects a diverse population. The employment of Lasso regression analysis allows for the identification of key predictive factors, ensuring a robust selection of variables. The model's high discrimination ability, indicated by area under the curve (AUC) values of 0.97 and 0.90 for training and validation sets respectively, demonstrates its potential clinical utility. Additionally, the study's calibration and decision curve analysis suggest that the nomogram could lead to significant improvements in clinical outcomes, making it a valuable tool for early identification and intervention in at-risk patients. Despite these advantages, the study does have some limitations. The reliance on a secondary data source may introduce biases associated with the dataset, including potential confounding variables that were not accounted for. Furthermore, the study's cross-sectional design limits the ability to infer causation between the identified predictors and the onset of sarcopenia. The sample size, while adequate for a preliminary analysis, may not fully capture the complexity of the relationship between stroke and sarcopenia across different demographic groups, particularly in underrepresented populations. Additionally, the clinical applicability of the nomogram in real-world settings requires further validation through prospective studies.

Future research should focus on several key areas. Longitudinal studies are needed to establish causative relationships between the identified risk factors and sarcopenia in stroke patients. Expanding the research to include diverse populations will enhance the nomogram's applicability across different demographics. Investigating the biological mechanisms underlying the relationship between stroke and sarcopenia could provide deeper insights into effective prevention and treatment strategies. Finally, exploring the integration of the nomogram into clinical practice, alongside patient outcomes, would be essential for assessing its real-world impact on healthcare delivery, and patient management.

## Conclusion

This study successfully developed and validated a nomogram for predicting sarcopenia risk in stroke patients based on key demographic and biochemical factors. The findings indicate that factors such as gender, BMI, and biochemical markers like ALP and hemoglobin significantly contribute to sarcopenia risk. By implementing this model in clinical practice, healthcare providers can identify at-risk patients earlier and tailor interventions to prevent or manage sarcopenia, thus enhancing overall recovery and quality of life for stroke survivors.

## Data Availability

Publicly available datasets were analyzed in this study. This data can be found here: https://www.cdc.gov/nchs/nhanes/index.htm.
