# Supplementary material for: Predicting sarcopenia risk in stroke patients: a comprehensive nomogram incorporating demographic, anthropometric, and biochemical indicators
Source: Front Neurol. 2024 Dec 9;15:1438575. doi: 10.3389/fneur.2024.1438575 (PMC11665213; doi:10.3389/fneur.2024.1438575)

The biochemical analysis and a comprehensive array of blood test markers

| **Variable** | **Non sarcopenia** | **Sarcopenia** | **P** |
| --- | --- | --- | --- |
|  | ***N=176*** | ***N=33*** |  |
| Gender: |  |  | 1.000 |
| Male | 73 (41.5%) | 14 (42.4%) |  |
| Female | 103 (58.5%) | 19 (57.6%) |  |
| Age | 48.7 (9.54) | 51.4 (7.69) | 0.074 |
| Race: |  |  | 0.003 |
| Mexican American | 18 (10.2%) | 5 (15.2%) |  |
| Other Hispanic | 9 (5.11%) | 6 (18.2%) |  |
| Non-Hispanic White | 62 (35.2%) | 16 (48.5%) |  |
| Non-Hispanic Black | 69 (39.2%) | 4 (12.1%) |  |
| Other Race | 18 (10.2%) | 2 (6.06%) |  |
| BMI | 30.1 (7.07) | 37.7 (9.44) | <0.001 |
| Weight | 85.6 (22.8) | 97.3 (27.1) | 0.025 |
| Hight | 168 (8.59) | 161 (9.54) | <0.001 |
| WAIST | 103 (17.2) | 116 (16.7) | <0.001 |
| Albumin(g/dL) | 4.13 (0.40) | 3.95 (0.36) | 0.011 |
| Albumin(g/L) | 41.3 (4.03) | 39.5 (3.57) | 0.011 |
| Alkaline Phosphatase (ALP) (IU/L) | 23.6 (13.0) | 24.2 (14.2) | 0.834 |
| Aspartate Aminotransferase (AST) (U/L) | 23.9 (10.7) | 22.2 (8.49) | 0.324 |
| Alkaline Phosphatase (ALP) (IU/L) | 77.2 (29.4) | 96.0 (51.7) | 0.049 |
| Blood Urea Nitrogen (mg/dL) | 13.8 (7.67) | 16.6 (14.2) | 0.271 |
| Blood Urea Nitrogen (mmol/L) | 4.91 (2.74) | 5.93 (5.08) | 0.272 |
| Total Calcium (mg/dL) | 9.36 (0.40) | 9.20 (0.34) | 0.020 |
| Total Calcium (mmol/L) | 2.34 (0.10) | 2.30 (0.09) | 0.020 |
| Creatine Phosphokinase (CPK) (IU/L) | 166 (160) | 101 (58.6) | <0.001 |
| Cholesterol(mg/dL) | 191 (46.0) | 195 (41.6) | 0.595 |
| Cholesterol(mmol/L) | 4.93 (1.19) | 5.04 (1.08) | 0.596 |
| Bicarbonate (mmol/L) | 25.1 (2.71) | 24.5 (2.28) | 0.161 |
| Creatinine(mg/dL) | 0.99 (0.58) | 1.20 (1.87) | 0.520 |
| Creatinine(umol/L) | 87.4 (51.2) | 106 (166) | 0.520 |
| Gamma Glutamyl Transferase (GGT) (IU/L) | 37.5 (42.3) | 45.8 (52.6) | 0.395 |
| Glucose(mg/dL) | 110 (45.3) | 125 (72.3) | 0.242 |
| Glucose(mmol/L) | 6.09 (2.51) | 6.95 (4.01) | 0.242 |
| Iron(ug/dL) | 80.8 (37.6) | 78.6 (30.7) | 0.724 |
| Iron(umol/L) | 14.5 (6.73) | 14.1 (5.50) | 0.720 |
| Lactate Dehydrogenase (LDH) (IU/L) | 141 (34.9) | 153 (47.1) | 0.195 |
| Phosphorus (mg/dL) | 3.69 (0.53) | 3.80 (0.68) | 0.398 |
| Phosphorus (mmol/L) | 1.19 (0.17) | 1.23 (0.22) | 0.398 |
| Total Bilirubin (mg/dL) | 0.55 (0.31) | 0.49 (0.29) | 0.310 |
| Total Bilirubin (umol/L) | 9.41 (5.36) | 8.39 (4.92) | 0.289 |
| Total Bilirubin (mg/dL) | 7.18 (0.51) | 7.07 (0.38) | 0.164 |
| Total Protein (g/L) | 71.8 (5.14) | 70.7 (3.84) | 0.164 |
| Uric acid (mg/dL) | 5.48 (1.65) | 5.59 (1.24) | 0.654 |
| Uric acid (umol/L) | 326 (98.3) | 333 (73.8) | 0.654 |
| Sodium (mmol/L) | 139 (2.72) | 139 (3.09) | 0.688 |
| Potassium (mmol/L) | 3.95 (0.41) | 4.05 (0.47) | 0.252 |
| Chloride (mmol/L) | 103 (3.60) | 102 (3.46) | 0.334 |
| Osmolality (mmol/Kg) | 279 (6.47) | 281 (9.22) | 0.188 |
| Globulin (g/dL) | 3.04 (0.54) | 3.12 (0.55) | 0.487 |
| Globulin (g/L) | 30.4 (5.42) | 31.2 (5.53) | 0.487 |
| Triglycerides(mmol/L) | 158 (106) | 194 (89.0) | 0.049 |
| Triglycerides(mmol/dL) | 1.79 (1.19) | 2.18 (1.00) | 0.049 |
| White blood cell count (1000 cells/uL) | 7.71 (2.34) | 8.52 (3.11) | 0.166 |
| Lymphocyte percent (%) | 30.0 (9.06) | 28.6 (9.49) | 0.419 |
| Monocyte percent (%) | 7.47 (2.15) | 7.81 (1.95) | 0.374 |
| Segmented neutrophils percent (%) | 58.9 (9.77) | 60.1 (10.2) | 0.545 |
| Eosinophils percent (%) | 2.84 (1.96) | 2.72 (1.70) | 0.728 |
| Basophils percent (%) | 0.83 (0.51) | 0.91 (0.33) | 0.263 |
| Lymphocyte number (1000 cells/uL) | 2.24 (0.76) | 2.28 (0.62) | 0.740 |
| Monocyte number (1000 cells/uL) | 0.56 (0.20) | 0.68 (0.41) | 0.107 |
| Segmented neutrophils num (1000 cell/uL) | 4.65 (1.95) | 5.28 (2.45) | 0.173 |
| Eosinophils number (1000 cells/uL) | 0.21 (0.15) | 0.23 (0.16) | 0.580 |
| Basophils number (1000 cells/uL) | 0.06 (0.06) | 0.08 (0.05) | 0.064 |
| Red blood cell count (million cells/uL) | 4.64 (0.56) | 4.76 (0.56) | 0.279 |
| Hemoglobin (g/dL) | 13.8 (1.80) | 14.2 (1.96) | 0.247 |
| Hematocrit (%) | 41.1 (4.92) | 42.2 (5.52) | 0.290 |
| Mean cell volume (fL) | 88.9 (7.16) | 88.8 (5.62) | 0.938 |
| Mean cell hemoglobin (pg) | 29.8 (2.94) | 29.8 (2.08) | 0.908 |
| Mean cell hemoglobin concentration (g/dL) | 33.4 (1.06) | 33.6 (0.84) | 0.407 |
| Red cell distribution width (%) | 14.1 (1.94) | 13.9 (1.02) | 0.342 |
| Platelet count (1000 cells/uL) | 248 (75.2) | 252 (79.5) | 0.781 |
| Mean platelet volume (fL) | 8.36 (0.92) | 8.60 (1.01) | 0.211 |


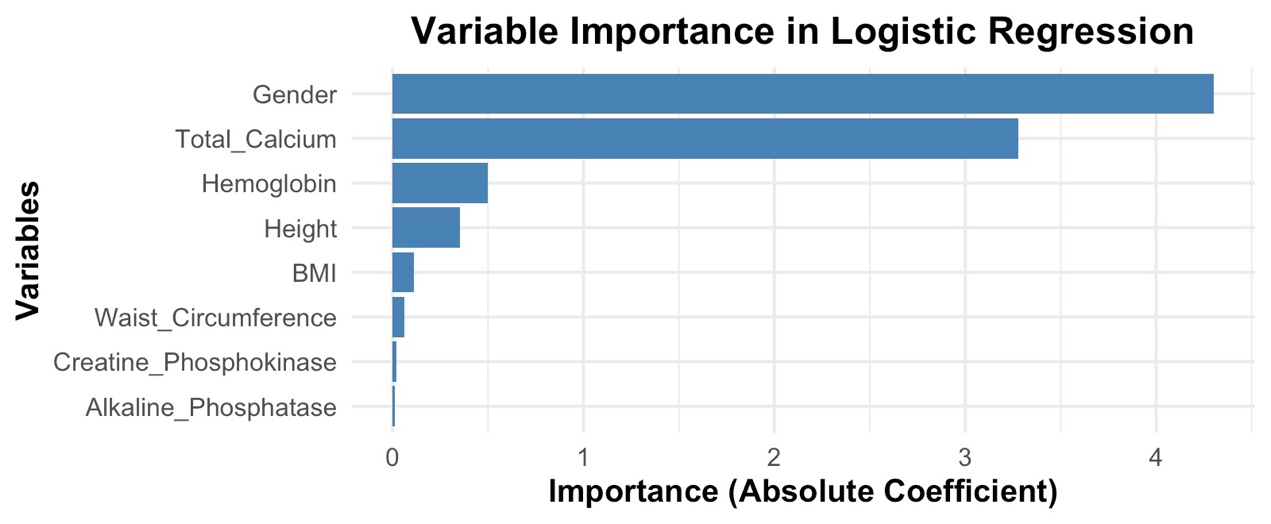


**Variables sorted by number of missings:**

Variable percentage Count

BMXWAIST 0.057416268 11

BMXBMI 0.004784689 1

BMXHT 0.000000000 0

RIAGENDR 0.000000000 0

LBXSAPSI 0.000000000 0

LBXSCA 0.000000000 0

LBXSCK 0.000000000 0

LBXHGB 0.000000000 0


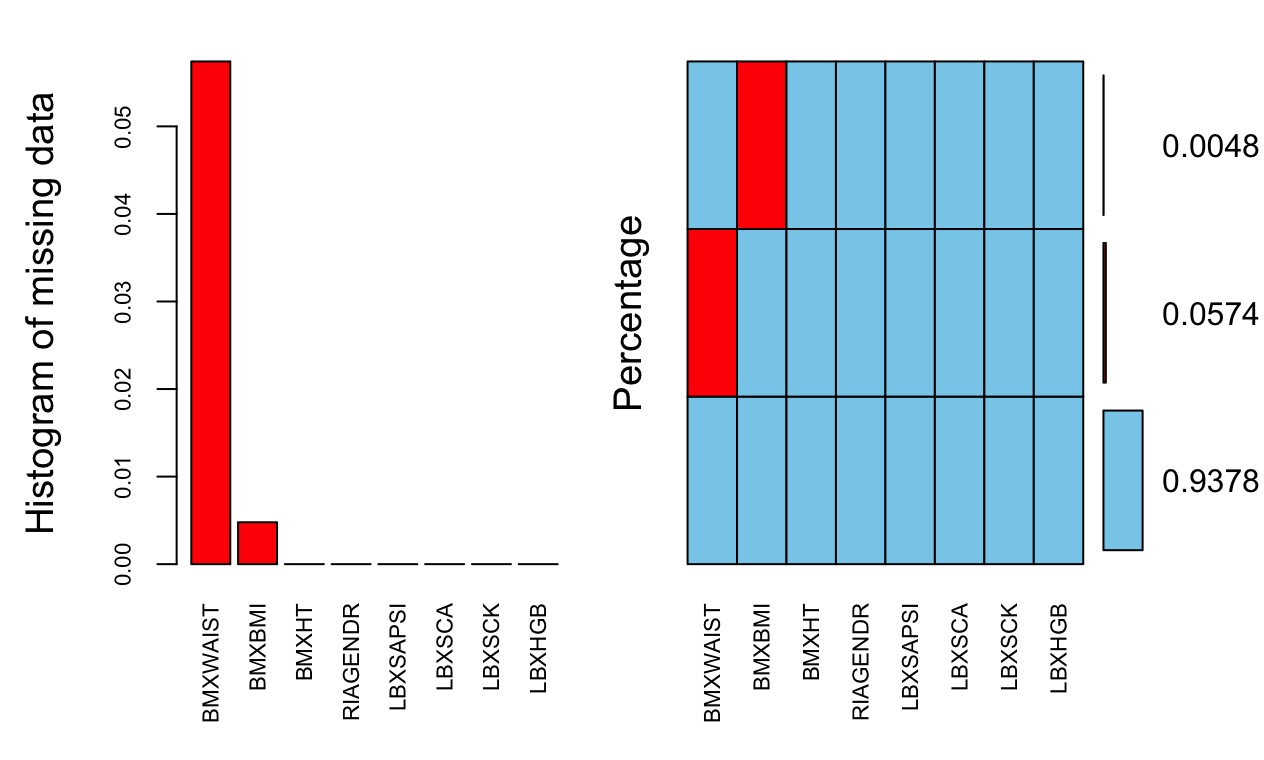

Supplement: Supplementary file 1 [file Data_Sheet_1.docx]
